# HDAd5/35^++^ Adenovirus Vector Expressing Anti-CRISPR Peptides Decreases CRISPR/Cas9 Toxicity in Human Hematopoietic Stem Cells

**DOI:** 10.1016/j.omtm.2018.04.008

**Published:** 2018-05-01

**Authors:** Chang Li, Nikoletta Psatha, Sucheol Gil, Hongjie Wang, Thalia Papayannopoulou, André Lieber

**Affiliations:** 1 Division of Medical Genetics, Department of Medicine, University of Washington, Box 357720, Seattle, WA 98195, USA; 2 Division of Hematology, Department of Medicine, University of Washington, Seattle, WA 98195, USA; 3 Department of Pathology, University of Washington, Seattle, WA 98195, USA

## Abstract

We generated helper-dependent HDAd5/35^++^ adenovirus vectors expressing CRISPR/Cas9 for potential hematopoietic stem cells (HSCs) gene therapy of β-thalassemia and sickle cell disease through re-activation of fetal γ-globin expression (HDAd-globin-CRISPR). The process of CRISPR/Cas9 gene transfer using these vectors was not associated with death of human CD34^+^ cells and did not affect their *in vitro* expansion and erythroid differentiation. However, functional assays for primitive HSCs, e.g., multi-lineage progenitor colony formation and engraftment in irradiated NOD/Shi-scid/interleukin-2 receptor γ (IL-2Rγ) null (NSG) mice, revealed toxicity of HDAd-globin-CRISPR vectors related to the prolonged expression and activity of CRISPR/Cas9. To control the duration of CRISPR/Cas9 activity, we generated an HDAd5/35^++^ vector that expressed two anti-CRISPR (Acr) peptides (AcrII4 and AcrII2) capable of binding to the CRISPR/Cas9 complex (HDAd-Acr). CD34^+^ cells that were sequentially infected with HDAd-CRISPR and HDAd-Acr engrafted at a significantly higher rate. Target site disruption frequencies in engrafted human cells were similar to those in pre-transplantation CD34^+^ cells, indicating that genome-edited primitive HSCs survived. *In vitro* differentiated HSCs isolated from transplanted mice demonstrated increased γ-globin expression as a result of genome editing. Our data indicate that the HDAd-Acr vector can be used as a tool to reduce HSC cytotoxicity of the CRISPR/Cas9 complex.

## Introduction

The CRISPR/Cas9 nuclease complex is composed of a single guide RNA (sgRNA) and the Cas9 nuclease. The sgRNA contains a 20-nt guide sequence that specifically binds to a genomic DNA target site. Target recognition by the CRISPR/Cas9 nuclease depends on the protospacer adjacent motif (PAM) sequence next to the DNA binding site. The Cas9 nuclease induces a blunt, double-stranded break (DSB) 3 bp upstream of the PAM sequence. The DSB is repaired by cellular enzymes creating insertions or deletions (indels) that disrupt the target site. The most widely used CRISPR Cas9 system is derived from *Streptococcus pyogenes* (SpCas9). Because it is thought that the CRISPR/Cas9 need to be expressed only for a short time to achieve permanent modification of the target genomic sequence, most of the delivery approaches focused on transient expression and activity of CRISPR/Cas9. These approaches include the electroporation with: (1) synthetic sgRNA and Cas9 protein complexes (ribonucleoproteins [RNPs]), (2) sgRNA and Cas9 mRNA, and (3) plasmids expressing sgRNA and Cas9. However, electroporation of peripheral blood-derived CD34^+^ cells can be associated with cytotoxicity.^1^, ^2^, ^3^ Alternative delivery strategies employing nano-particles or virus-mediated delivery have been recently explored, with viral delivery being the optimal vehicle for certain applications enhancing efficiency while minimizing toxicity.^4^, ^5^ Furthermore, viral delivery of the desired nuclease can be also applicable for *in vivo* hematopoietic stem cell (HSC) genome editing.^6^

We have successfully used non-integrating adenovirus vectors for gene transfer into CD34^+^ cells. Because commonly used species C adenovirus (Ad) serotype 5-based vectors do not efficiently transduce CD34^+^ cells, we developed chimeric Ad5 vectors that carry fibers from species B Ad serotype 35 (Ad5/35). These vectors target CD46, a membrane protein that is uniformly expressed on human CD34^+^ cells.^7^ We and others have shown that Ad5/35 vectors efficiently transduce HSCs, including quiescent HSCs, *in vitro*.^8^, ^9^, ^10^ These studies were performed with first-generation Ad5/35 vectors that contain viral genes that are expressed in transduced cells and affect HSC viability and function.^11^ To avoid HSC cytotoxicity associated with first-generation Ad5/35 vectors, we generated helper-dependent HDAd5/35^++^ vectors devoid of all viral genes. These vectors also contained a series of mutations in the Ad35 fiber knob that increased the affinity to CD46 more than 25-fold and allowed for more efficient cell transduction at lower MOIs.^12^

The production of HDAd5/35^++^ vectors involves the co-transfection of a plasmid containing the helper-dependent Ad (HDAd) vector genome and an Ad5/35^++^ helper virus that provides all structural and non-structural viral proteins but cannot be packaged. In contrast with recombinant adeno-associated virus (rAAV) and lentivirus vectors, HDAd5/35^++^ vector production does not require large-scale plasmid transfection and routinely yields ∼1 × 10^13^ viral particles (vp) per preparation. The purified virus preparation can then be used as a stock for further large-scale production that makes HDAd5/35^++^ a cost-efficient alternative vector platform for gene therapy, specifically for *in vivo* HSC gene therapy.^6^, ^7^, ^13^

In previous studies with HAd5/35^++^ vectors expressing a zinc-finger nuclease (ZFN), we found that transduced CD34^+^ cells only poorly engraft in irradiated NOD/Shi-scid/interleukin-2 receptor γ (IL-2Rγ) null (NSG) mice.^14^, ^15^ This was not due to the HDAd5/35^++^ transduction process, because engraftment rates were comparable with untransduced cells with a GFP-expressing HDAd5/35^++^ vector. We therefore speculated that this is related to ZFN expression over an extended time period. In the present study, we encountered a similar problem with HDAd5/35^++^ vectors expressing CRISPR/Cas9. We therefore explored the potential of naturally occurring CRISPR/Cas9 inhibitor peptides to regulate the duration of CRISPR/Cas9 activity after HDAd5/35^++^ delivery into CD34^+^ cells. CRISPR systems protect bacteria against invading bacteriophages. In response to this, phages have evolved proteins (anti-CRISPRs [Acr]) that bind to and inactivate Cas proteins as they search for foreign nucleic acid.^16^ In our study, we focused on AcrIIA2 and A4.^17^ These peptides have a length of 87 amino acids (aa) and are active against a broad spectrum of Cas9 orthologs including spCas9. AcrIIA4 binds to a region of Cas9 that normally engages the PAM, and thus prevents DNA cutting.^17^, ^18^, ^19^ In addition, it blocks target DNA access to key catalytic domains of Cas9.^19^, ^20^, ^21^ Because Acr can inactivate CRISPR/Cas9 they could provide an efficient off switch for Cas9-based applications.

Here we studied whether timed expression of AcrIIA2 and AcrIIA4 from an HDAd5/35^++^ vector can modulate CRISPR/Cas9 activity in CD34^+^ cells and decrease CRISPR/Cas9-associated toxicity to HSCs.

## Results

### HDAd-CRISPR Vectors

**Figure 1:**
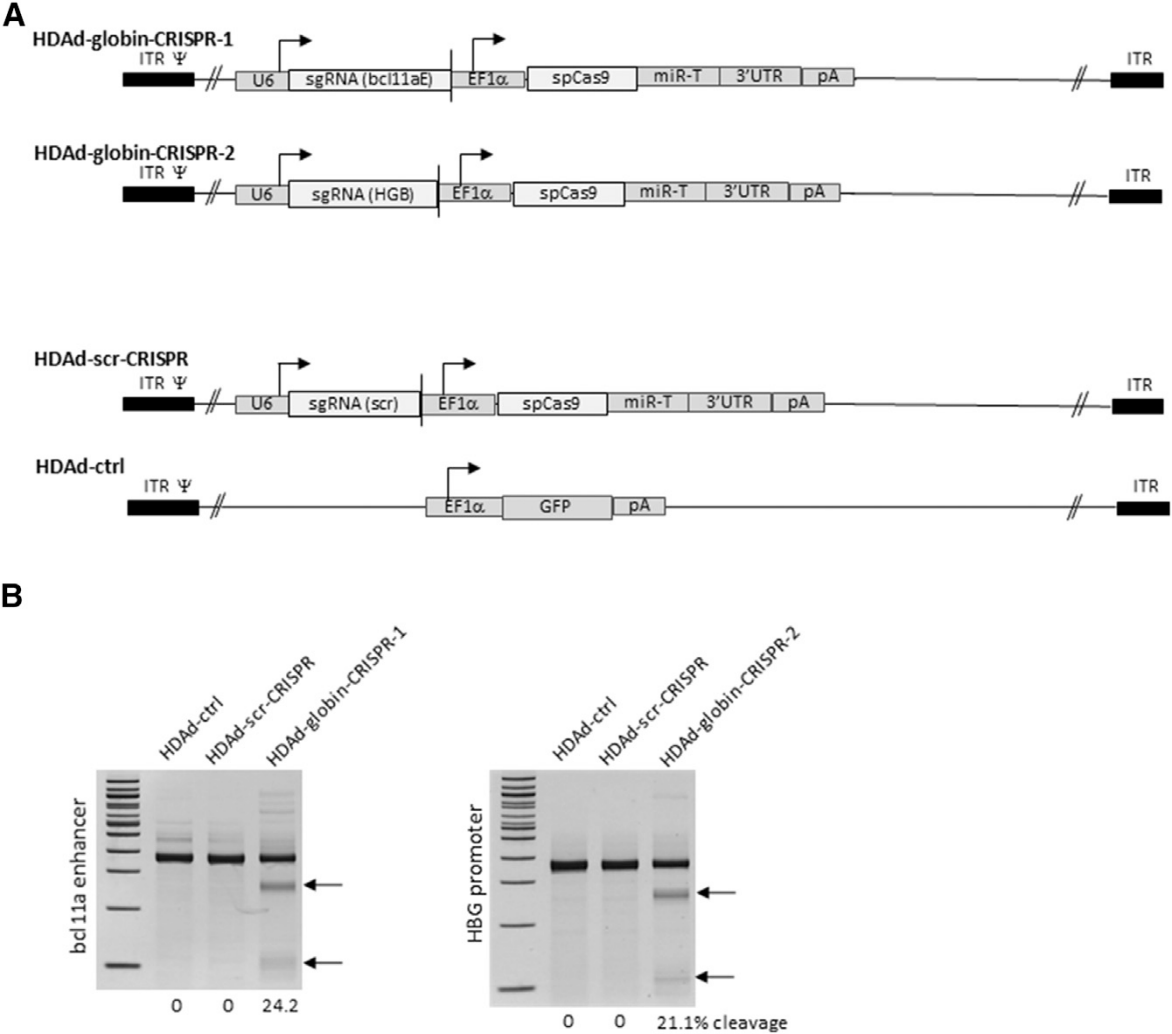
HDAd5/35^++^ Vectors Expressing CRISPR/Cas9 (A) Vector structure. HDAd-globin-CRISPR-1, HDAd-globin-CRISPR-2, and HDAd-scr-CRISPR contained the sgRNA gene transcribed by PolIII from the U6 promoter and the spCas9 gene under the control of the EF1α promoter. Cas9 expression is controlled by miR-183-5p and miR-218-5p, which suppress Cas9 expression in HDAd producer 116 cells, but do not negatively affect Cas9 expression in CD34^+^ cells.^14^ The corresponding microRNA (miRNA) target sites were embedded into the 3′ UTR of the β-globin gene. HDAd-globin-CRISPR-1 targets the erythroid bcl11a enhancer. HDAd-globin-CRISPR-2 targets a site in the human γ-globin (HBG) promoter. HDAd-scr-CRISPR expressed a scrambled guide RNA and has no target in the human genome. HDAd-ctr (without CRISPR/Cas9) is used to assess cytotoxicity of HDAd5/35^++^ transduction. (B) Validation of target site cleavage. CD34^+^ cells were infected with the indicated vectors at an MOI of 2,000 vp/cell under low cytokine and growth factor concentrations. Genomic DNA was isolated 3 days later and analyzed by T7E1 nuclease assay with primers specific to the bcl11a-enhancer region for analysis of HDAd-globin-CRISPR-1 (left panel) and the HBG region for analysis of HDAd-globin-CRISPR-2 (right panel). Bands corresponding to cleaved DNA are marked by arrows. The numbers below the lanes indicate the percentage of disrupted alleles.

We generated two HDAd5/35^++^ CRISPR/Cas9 vectors capable of reactivation of fetal γ-globin for potential gene therapy of β-thalassemia and sickle cell disease. The first vector expressed a CRISPR/Cas9 targeted to the erythroid enhancer of the known γ-globin suppressor BCL11A^22^, ^23^ (Figure 1A, HDAd-globin-CRISPR-1). The second vector was specific to a region in the γ-globin promoter that contains a recently identified binding site for an isoform of BCL11A^24^ (Figure 1A, HDAd-globin-CRISPR-2). To discriminate HSC cytotoxicity caused by the DNA-bound sgRNA/Cas9 complex mediating DSBs from other effects of the Cas9 protein or sgRNA/Cas9 complex, we also constructed a vector that expressed a scrambled guide RNA without homology in the human genome^25^ (Figure 1A, HDAd-scr-CRISPR). An HDAd5/35^++^ without sgRNA and Cas9 was used as a control (Figure 1A, HDAd-ctrl).

To validate the activity of the HDAd-globin-CRISPR vectors, we transduced CD34^+^ cells and analyzed 2 days later target site-specific disruption using a mismatch-sensitive T7E1 assay (Figure 1B). We found that 24.2% and 21.2% of the corresponding targeted sites were cleaved by the bcl11a enhancer-specific and HBG promoter-specific CRISPRs, respectively. The HDAd-scr-CRISPR vector did not cleave the target sites.

### *In Vitro* Cytotoxicity Studies with Transduced CD34^+^ Cells

**Figure 2:**
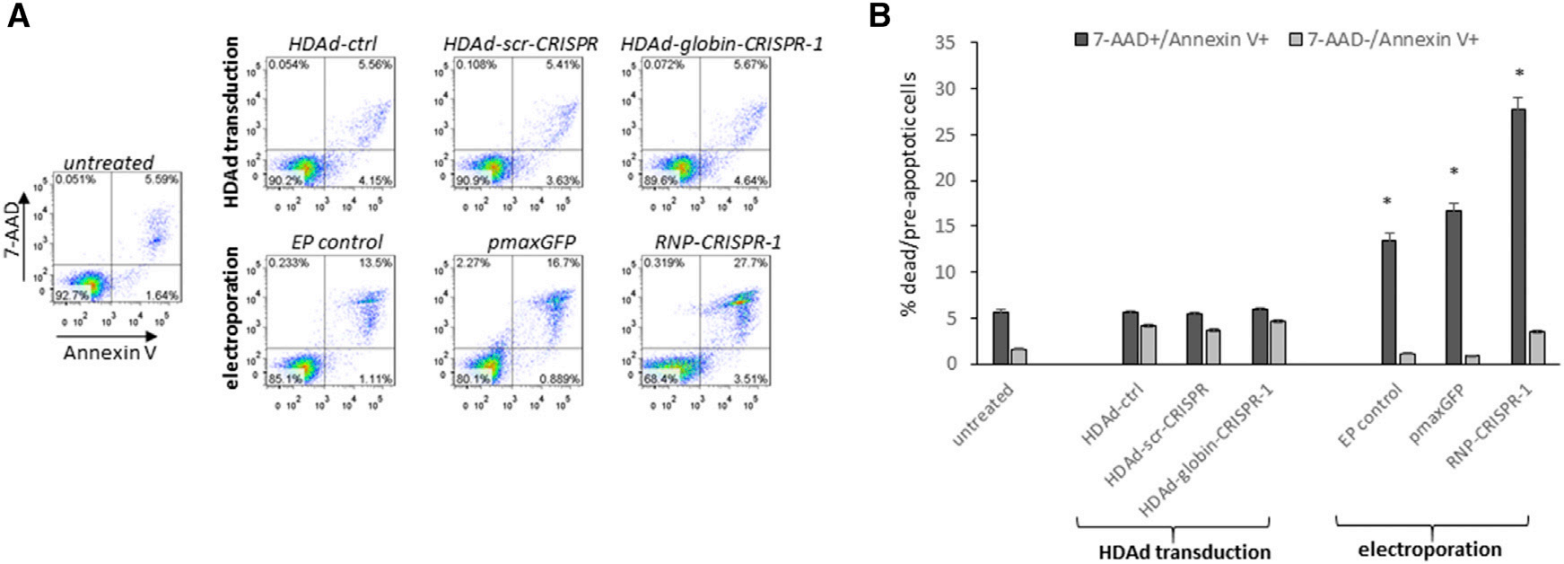
Cytotoxicity Associated with the Gene Transfer by HDAd5/35^++^ Vector or Electroporation (A) CD34^+^ cells were either transduced with HDAd5/35^++^ (upper panels) or electroporated (lower panels). Cell death by apoptosis or necrosis was measured by Annexin V/7-AAD flow cytometry 18 hr after adding the vectors or after electroporation. The left panel shows untreated cells. 7-AAD^+^/Annexin V^+^ cells are necrotic and dead. 7-AAD^−^/Annexin V^+^ cells are pre-apoptotic cells. CD34^+^ cells were infected with HDAd-ctrl, HDAd-scr-CRISPR, or HDAd-globin-CRISPR-1 at an MOI of 2,000 vp/cells. Nucleofection by electroporation was performed with PBS (“EP-control”), the plasmid pmaxGFP, or the globin CRISPR-1 RNP (see Materials and Methods for details). (A) Flow cytometry for representative samples. (B) Summary of data. N = 3. *p < 0.05 compared with untreated.

In agreement with previous studies,^14^ the process of transduction of CD34^+^ cells with HDAd5/35^++^ vectors (regardless of CRISPR/Cas9 expression) did not cause cell death as reflected by 7-aminoactinomycin D (7-AAD)^+^/Annexin V^+^ cells at 18 hr after adding the vectors at an MOI of 2,000 vp/cell (Figures 2A and 2B). In contrast, the process of CD34^+^ cell electroporation, particularly the electroporation with a globin-CRISPR-1 RNP, was associated with significant cell death.

To assess cytotoxicity in HSCs and early progenitors over longer time periods, we employed two functional *in vitro* assays: (1) the ability to differentiate into erythroid cells in liquid culture *in vitro*, and (2) the ability to form multi-lineage progenitor colonies in semi-solid methyl-cellulose medium.

**Figure 3:**
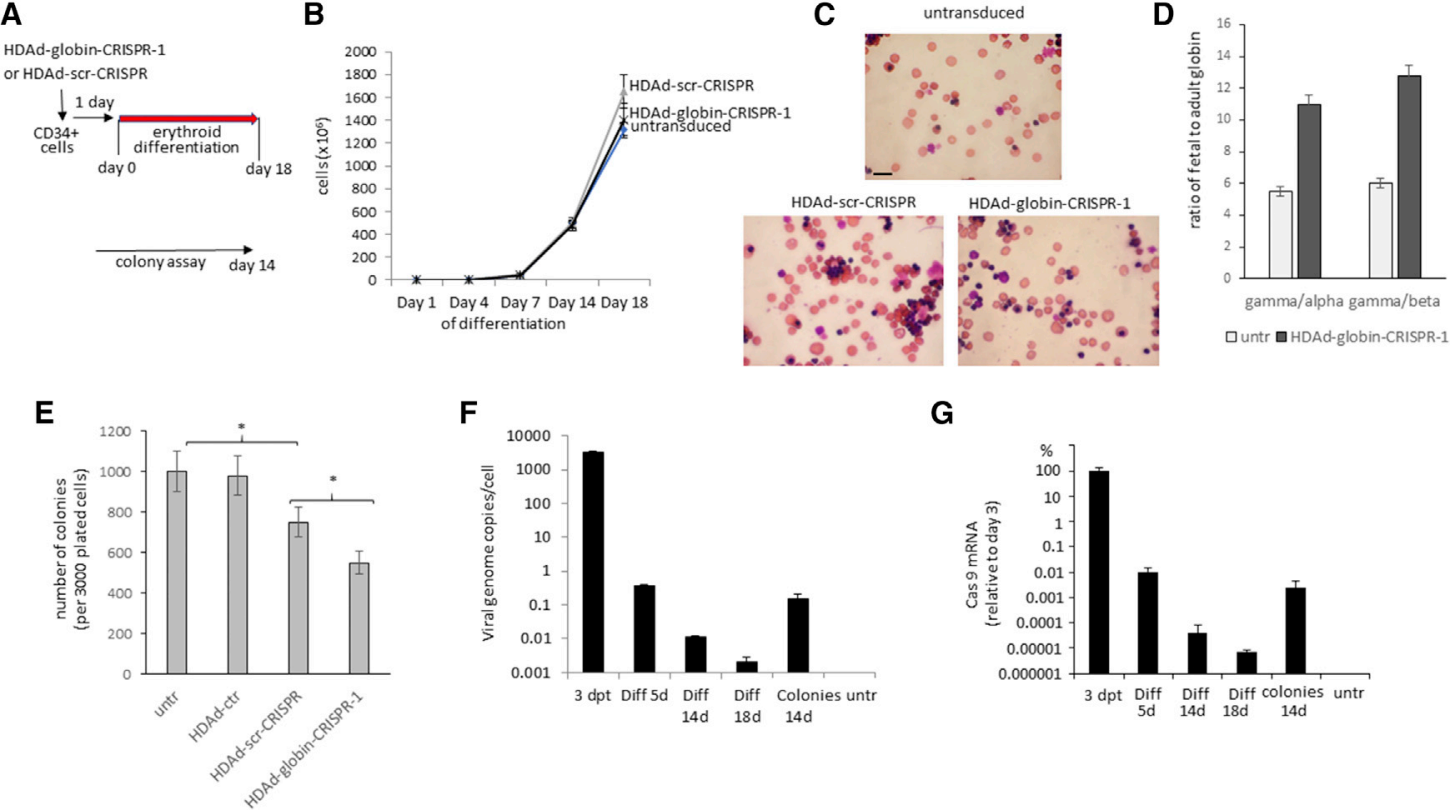
*In Vitro* Cytotoxicity of HDAd-globin-CRISPR Vectors on Erythroid Progenitors and Colony-Forming Cells (A) Schematic of *in vitro* assays. Upper panel: erythroid expansion and differentiation assay. Twenty-four hours after transduction of CD34^+^ cells at an MOI of 2,000 vp/cell in low-cytokine medium, cells were subjected to a three-step differentiation procedure over 18 days (see Materials and Methods). Lower panel: multilineage progenitor colony assay. Twenty-four hours after transduction, colonies were plated in methylcellulose supplemented with cytokines and growth factors. Colonies with more than 100 cells were scored 14 days later. (B) Growth kinetics during erythroid expansion and differentiation. At the indicated time points viable cells were counted by trypan blue exclusion. N = 3. The difference between the curves is not significant. (C) Representative microphotographs of cultures at day 18 of differentiation. Visible are red cells without nuclei, pyrenocytes, and less differentiated cells with purple-stained nuclei. The scale bar represents 20 μm. (D) Globin HPLC. Lysates from differentiated cells were subjected to reverse-phase HPLC, and the amount of α-, β-, and γ-globin was measured. Shown is the ratio of fetal γ- to adult α- and β-globin for untransduced cells and HDAd-globin-CRISPR-1-transduced cells. (E) Progenitor colony assay. Twenty-four hours after transduction of CD34^+^ cells with the indicated vectors, 3,000 cells were mixed with methylcellulose and plated in 35-mm dishes. Colonies were counted 14 days later. (F) Number of HDAd-globin-CRISPR-1 DNA genomes per cell. Genomic DNA from the indicated samples was subjected to qPCR with Cas9-gene-specific primers. N = 3. (G) Quantification of Cas9 mRNA. Total RNA was subjected to qRT-PCR with Cas9-specific primers. mRNA levels at day 3 after infection of CD34^+^ cells with HDAd-globin-CRISPR-1 were taken as 100%. N = 3.

To evaluate cytotoxicity to early erythroid progenitors, we subjected CD34^+^ cells 24 hr after transduction with HDAd-globin-CRISPR-1 or HDAd-scr-CRISPR to erythroid expansion and differentiation in medium that contained stem cell factor (SCF), IL-3, and erythropoietin (Epo)^26^ (Figure 3A, upper panel). The total period of the three-step differentiation process is 18 days. During this time, no significant difference in the growth kinetics was observed between untransduced, HDAd-globin-CRISPR-1, and HDAd-scr-CRISPR-transduced cells (Figure 3B). At the end of the differentiation period, all three cultures contained comparable numbers of enucleated red cells (Figure 3C). We also measured the therapeutic effect of HDAd-CRISPR-1, i.e., the reactivation of γ-globin, in differentiated cells by high-performance liquid chromatography (HPLC) that can quantitate adult (α- and β-) and fetal (γ-) globin (Figure 3D). The ratio of fetal to adult globin was more than doubled, indicating a reverse switch from adult to fetal globin because of the disruption of the erythroid bcl11a enhancer.

In the second assay, the effect on colony-forming units (CFUs) within CD34^+^ cells was analyzed (Figure 3A, lower panel). In methylcellulose supplemented with cytokines and growth factors, CFUs are able to give rise to multi-lineage progenitor colonies. CFUs are therefore more primitive than the erythroid progenitors assayed in the previous study. Colonies were counted at day 14 after plating transduced CD34^+^ cells. In agreement with previous data,^11^ CD34^+^ cell transduction with a GFP-expressing HDAd vector (HDAd-ctr) had no effect on colony formation. In contrast, the number of colonies was significantly decreased in HDAd-globin-CRISPR-1 and HDAd-scr-CRISPR samples (Figure 3E). There were significantly fewer colonies in the setting that expressed a DNA-cleaving CRISPR/Cas9 complex (HDAd-globin-CRISPR-1) compared with HDAd-scr-CRISPR-transduced samples. This indicates that CFU cytotoxicity appears to be mediated by DSBs, as well as other negative effects of the scr-sgRNA, Cas9, or scr-sgRNA/Cas9 complex on CFUs.

To further investigate CRISPR/Cas9-mediated toxicity, we measured HDAd-globin-CRISPR-1 vector copy numbers by qPCR using Cas9-specific primers (Figure 3F). Vector copy number rapidly declined in fast proliferating cells more than 5 orders of magnitude during erythroid *in vitro* differentiation (compared with vector copies present at day 3 after transduction). However, in colonies (consisting of 200–500 cells), the initially transduced CD34^+^ cells underwent fewer (8–9) rounds of cell division, and remaining vector DNA levels were higher. A similar kinetics was seen for Cas9 mRNA measured by qRT-PCR (Figure 3G). This implies that episomal HDAd vector DNA and Cas9 expression is maintained longer in slowly proliferating cells. We speculate that this extended Cas9 expression contributed to cytotoxicity observed in the CFU assay.

### *In Vivo* Engraftment Studies with Transduced CD34^+^ Cells

**Figure 4:**
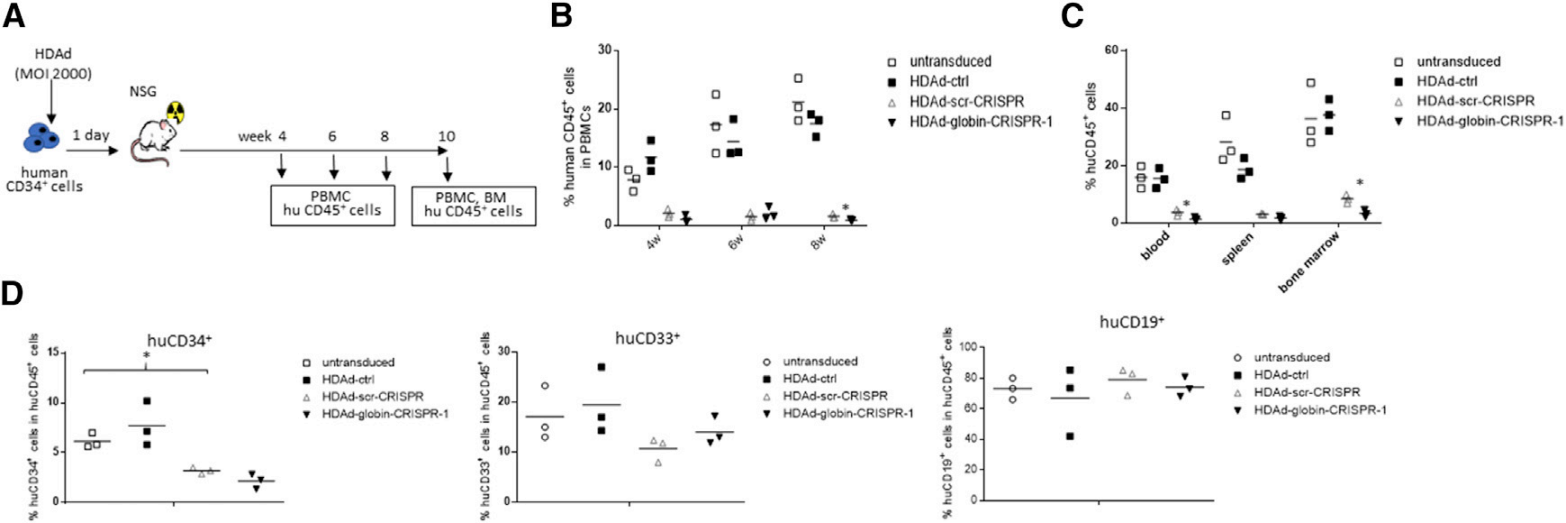
*In Vivo* Engraftment with HDAd-CRISPR-Transduced CD34^+^ Cells (A) Schematic of experiment. CD34^+^ cells were cultured overnight under low cytokine concentration conditions and transduced with HDAd-ctrl, HDAd-scr-CRISPR, or HDAd-globin-CRISPR-1 at an MOI of 2,000 vp/cell. Twenty-four hours later, one million transduced cells were transplanted into sublethally irradiated NSG mice. (B) The percentage of human CD45^+^ leukocytes in white blood cells of blood samples collected at weeks 4, 6, and 8 was measured by flow cytometry to assess engraftment. Each symbol is an individual animal. N = 3. (C) At week 10 after transplantation, mice were sacrificed and the percentage of huCD45^+^ cells was analyzed in blood, spleen, and bone marrow. (D) In bone marrow huCD45^+^ cells, the percentages of human CD34^+^ HSCs, CD19^+^ lymphoid cells, and CD33^+^ myeloid cells were measured by flow cytometry. *p < 0.05.

The optimal assay for demonstrating the transduction of HSCs within CD34^+^ cells involves the transplantation of cells into sublethally irradiated NSG mice (Figure 4A). HSCs engraft, expand, and undergo limited differentiation in these mice. Engraftment rates of human CD45^+^ (huCD45^+^) cells measured at weeks 4, 6, and 8 after transplantation were in the range of 10%–20% in mice transplanted with untransduced CD34^+^ cells and with CD34^+^ cells transduced with HDAd-ctrl (Figure 4B). This is in agreement with our previous studies^11^, ^14^, ^27^ and suggests that HDAd5/35^++^ transduction does not reduce the engraftment potential of CD34^+^ cells. In contrast, engraftment of HDAd-scr-CRISPR- and HDAd-globin-CRISPR-1-transduced cells was less than 2%. This was confirmed in blood, spleen, and bone marrow samples harvested at week 10 after transplantation (Figure 4C). While both CRISPR vectors decreased engraftment, the effect was significantly stronger for HDAd-globin-CRISPR-1 in blood and bone marrow. Within the engrafted human cells in the bone marrow, CD34^+^ HSCs were most affected by HDAd-scr-CRISPR- and HDAd-globin-CRISPR-1 transduction, whereas this effect was less pronounced for myeloid (CD33^+^) and lymphoid (CD19^+^) cells (Figure 4D). Because of the low engraftment rates of HDAd-globin-CRISPR-1-transduced CD34^+^ cells, analysis of a potential therapeutic effect based on γ-globin expression in human erythroid cells was not possible.

Our *in vivo* study confirms that CRISPR/Cas9 exerts cytotoxicity to HSCs, specifically to NSG-repopulating CD34^+^ cells. In these cytotoxicity studies, we focused on HDAd-globin-CRISPR-1. Key findings (effect of CFUs and NSG-engrafting cells) were reproduced with HDAd-globin-CRISPR-2 and in CD34^+^ cells from a second donor (data not shown).

### Inhibition of CRISPR/Cas9 Cleavage by HDAd-Acr in HUDEP-2 Cells

**Figure 5:**
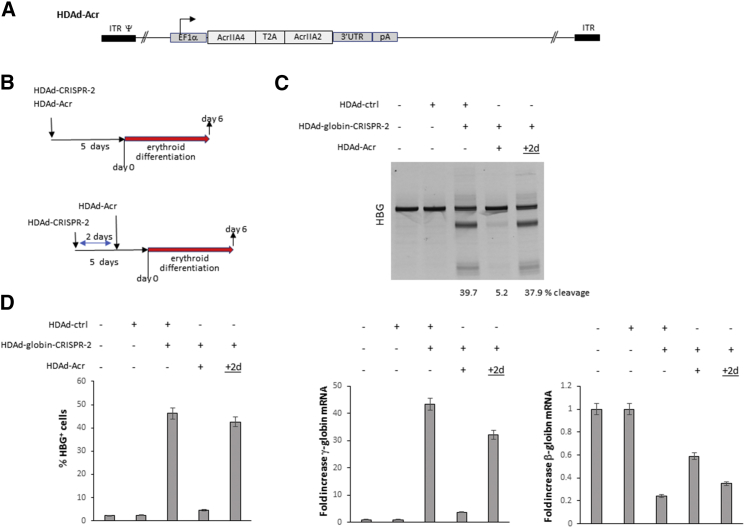
Inhibition of CRISPR/Cas9 Cleavage Activity by Sequential Transduction with HDAd-globin-CRISPR-2 and HDAd-Acr Vectors in HUDEP-2 Cells (A) The HDAd-Acr vector contains the ORFs for AcrIIA4 and AcrIIA2 linked through a self-cleaving picornavirus-derived T2A peptide. The bovine growth hormone gene polyadenylation signal (PA) is used to terminate transcription from the EF1α promoter. (B) Schematic of the two infection regimens (description in text). The MOI used for all vectors was 500 vp/cell. (C) T7E1 assay with genomic DNA isolated from cells at the end of erythroid differentiation. “+2d” implies that the sequential infection regimen was used with a 2-day interval between the two vectors. A representative sample is shown. (D) Analysis of γ- and β-globin expression at the end of erythroid differentiation. Left panel: γ-globin flow cytometry. Middle panel: fold increase of γ-globin mRNA over untransduced samples. N = 3. Right panel: fold increase of adult β-globin mRNA over untransduced samples. N = 3.

To control the duration of CRISPR/Cas9 activity, we generated an HDAd5/35^++^ vector expressing two Acr (AcrIIA4 and AcrIIA2)^17^ under control of the EF1α promoter (HDAd-Acr) (Figure 5A). This is the same promoter used for Cas9 expression in the HDAd-globin-CRISPR vectors and, therefore, should provide comparable expression levels of Cas9 and Acr. The ability of HDAd-Acr to block CRISPR/Cas9 cleavage was first tested in Human Umbilical cord blood-Derived Erythroid Progenitor (HUDEP-2) cells.^28^ Upon culture in erythroid differentiation media, HUDEP-2 cells closely resemble adult erythroid cells because they express predominantly β-globin. In our study, we used a co-infection approach with HDAd-globin-CRISPR-2 and HDAd-Acr at an MOI that allows for efficient co-infection with both vectors.^7^, ^14^

We used two transduction regimens (Figure 5B). Simultaneous co-infection of both vectors should lead to co-expression of both CRISPR/Cas9 and Acr, and block cleavage activity. Infection with the HDAd-Acr vector 2 days after the HDAd-CRISPR vector should allow CRISPR/Cas9 to exert its cleavage activity and, considering the outcome of regimen 1, should block CRISPR/Cas9 activity after day 2. The transduction was embedded in a 5-day pre-differentiation period, followed by erythroid differentiation for 6 days. At the end of differentiation, target site cleavage and γ-globin reactivation were measured. Untransduced or HDAd-ctrl transduced HUDEP-2 cells displayed no cleavage of the bcl11a-enhancer target site. HDAd-globin-CRISPR-1 infection resulted in 40% (±5%) cleavage (Figure 5C). Co-infection with HDAd-Acr decreased cleavage 8- to 9-fold. Infection with HDAd-Acr 2 days after the HDAd-CRISPR vector resulted in a cleavage frequency comparable with HDAd-globin-CRISPR-1-only infection. Inhibition of CRISPR/Cas activity by HDAd-Acr was confirmed by measuring γ-globin expression levels by flow cytometry (Figure 5D, left panel) or globin qRT-PCR (Figure 5D, middle and right panels). γ-Globin expression was correlated with the editing levels. As expected, disruption of the erythroid bcl11a enhancer resulted in a reverse γ-to-β-globin switch as reflected by an increase in γ-globin mRNA (Figure 5D, middle panel) and a decrease in β-globin mRNA levels (Figure 5D, right panel) in edited cells. Transduction and T7E1 nuclease studies with HDAd-globin-CRISPR-1 yielded similar results (data not shown).

Overall, these studies indicate that timed transduction with HDAd-CRISPR-globin and HDAd-Acr is capable to block CRISPR/Cas9 activity after the complex has completed target site cleavage.

### *In Vivo* Studies with Transplanted CD34^+^ Cells that Were Sequentially Transduced with HDAd-globin-CRISPR and HDAd-Acr Vectors

**Figure 6:**
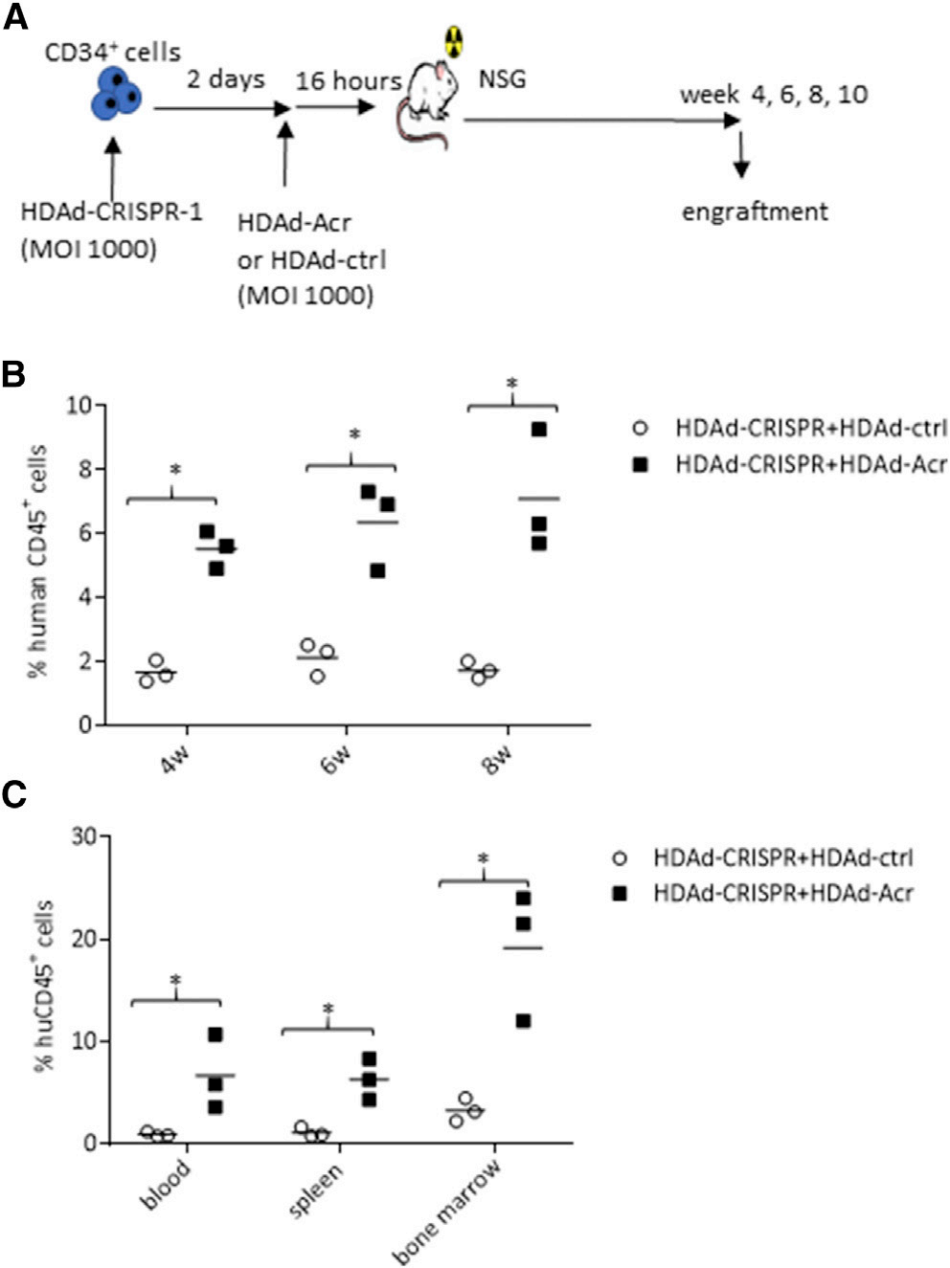
Engraftment of CD34^+^ Cells that Were Subsequently Transduced with HDAd-CRISPR-1 and HDAd-Acr (A) Schematic of the experiment. CD34^+^ cells were transduced in low-cytokine medium with HDAd-CRISPR-1 at an MOI of 2,000 vp/cell. Two days later, cells were infected with HDAd-Acr at the same MOI. Sixteen hours after the last infection, CD34^+^ cells were transplanted, and engraftment was measured. (B) Percentage of human CD45^+^ cells in PBMC samples measured at weeks 4, 6, and 8 after transplantation. (C) Percentage of human CD45^+^ cells in PBMCs, splenocytes, and bone marrow mononuclear cells measured at week 10 after transplantation. *p < 0.05.

In the first experiment, CD34^+^ cells were transduced with the HDAd5/35^++^ CRISPR-1 vector (Figure 6A). Two days later, the culture was infected with HDAd-Acr at an MOI that should provide the infection of the majority of HDAd-CRISPR-1-transduced cells to block further CRISPR/Cas9 activity. As a negative control for Acr activity, CD34^+^ cells were subsequently infected with HDAd-CRISPR-1 and the control vector HDAd-ctrl. Sixteen hours after infection with the second virus, cells were transplanted into irradiated NSG mice and engraftment was measured as described above for Figure 4.

**Figure 7:**
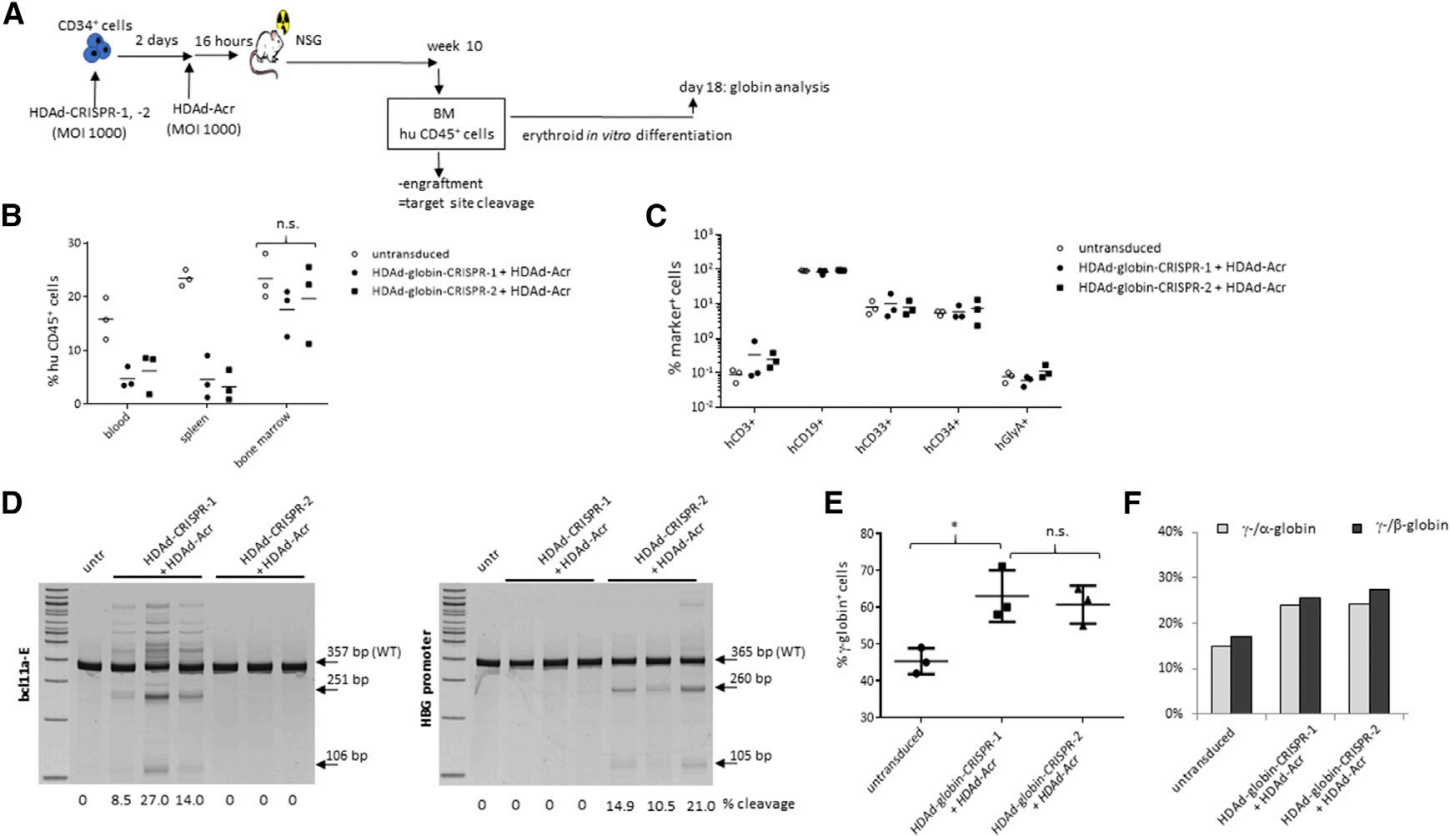
Engraftment and γ-Globin Reactivation Studies with HDAd-globin-CRISPR-1 and HDAd-globin-CRISPR-2 + HDAd-Acr (A) Schematic of the experiment. CD34^+^ cells were transduced with HDAd-CRISPR/Acr as described in Figure 6. Untransduced cells were incubated for 2 days + 16 hr in the same medium as transduced cells. At week 10 after transplantation, in addition to engraftment studies, human CD45^+^ cells were isolated from the bone marrow by magnetic-activated cell sorting (MACS) and subjected to T7E1 assay and erythroid *in vitro* differentiation for globin analysis by flow cytometry and HPLC. (B) Percentage of huCD45^+^ cells in tissues. (C) Percentage of human CD3^+^ (T cell lymphoid), CD19^+^ (B cell lymphoid), CD33^+^ (myeloid), CD34^+^ (HSCs), and GlyA^+^ (erythroid) cells in bone marrow CD45^+^ cells. (D) Bone marrow human CD45^+^ cells were subjected to T7E1 nuclease assay with primers specific to the bcl11a-enhancer region for analysis of HDAd-globin-CRISPR-1 (left panel) and the HBG region for analysis of HDAd-globin-CRISPR-2 (right panel). (E) γ-Globin flow cytometry of cells after erythroid *in vitro* differentiation. (F) Ratio of γ-globin to adult α- or β-globin measured after erythroid differentiation by HPLC. For this analysis the samples from three mice per group were pooled. *p < 0.05.

In the blood, at week 8 post-transplantation, the percentage of huCD45^+^ cells was 3- to 4-fold higher in animals that received the HDAd-CRISPR-1 + HDAd-Acr-transduced cells compared with the control setting (Figure 6B). A similar increase in engraftment by HDAd-Acr was seen in blood, spleen, and bone marrow of mice sacrificed at week 10 after transplantation (Figure 6C). Based on these data, we performed a second, more detailed study with HDAd-globin-CRISPR-1 and HDAd-globin-CRISPR-2 in which we also evaluated target site disruption and γ-globin reactivation (Figure 7A). As in the study shown in Figure 5, CD34^+^ cells were infected with HDAd-Acr 2 days after HDAd-globin-CRISPR-1/-2 transduction. Analysis of engraftment in tissues at week 10 after transplantation demonstrated ∼5% huCD45^+^ cells in PBMCs and splenocytes, and ∼20% huCD45^+^ cells in the bone marrow for both HDAd-CRISPR vectors (Figure 7B). Notably, engraftment rates in the bone marrow were not significantly different in mice transplanted with untransduced and HDAd-CRISPR/Acr-transduced cells. Differences in blood and spleen could be because of residual CRISPR/Cas9 activity that negatively affects the exit of differentiated cells from the bone marrow. Analysis of subfractions in bone marrow engrafted huCD45^+^ cells showed no difference in the frequency of human CD3^+^, CD19^+^, CD33^+^, CD34^+^, and GlyA (erythroid) cells between mice that received untransduced CD34^+^ cells or cells transduced with HDAd-globin-CRISPR-1/-2 + HDAd-Acr (Figure 7C). This is in contrast to the HDAd-CRISPR-1 (only) transduction study shown in Figure 4, where the percentage of CD34^+^ cells was dramatically reduced. At week 10, engrafted huCD45^+^ cells were isolated from the bone marrow, and target site cleavage was measured by T7E1 assay (Figure 7D). In mice transplanted with HDAd-globin-CRISPR-1 cells + HDAd-Acr transduced cells, bcl11a-enhancer target site cleavage ranged from 8.5% to 27% in individual mice. The HDAd-globin-CRISPR-2 + HDAd-Acr samples showed HBG-promoter target site cleavage ranging from 10.5% to 21%. These cleavage frequencies were comparable with pre-transplantation frequencies (data not shown and Figure 1B). When bone marrow huCD45^+^ cells were differentiated into erythroid cells, significantly higher percentages of γ-globin^+^ cells were detected in HDAd-globin-CRISPR+HDAd-Acr samples compared with samples from mice that were transplanted with untransduced cells. Furthermore, the ratio of γ-globin to adult α- and β-globin mRNA was increased compared with untransduced cells, indicating a partial switch from adult globin to fetal γ-globin.

Overall, our studies in the NSG transplantation model demonstrated that CD34^+^ cell transduction with HDAd-Acr after completion of Cas9-sgRNA-based gene editing can shorten the duration that CRISPR/Cas9 is active and consequently decrease cytotoxicity to HSCs, thus enabling more efficient engraftment and survival of gene-edited HSCs.

## Discussion

Among the advantages of using HDAd5/35^++^ vectors for delivery of CRISPR/Cas9 expression cassettes are the ability to transduce non-dividing primitive HSCs, low HSC cytotoxicity associated with the gene transfer process, the large insert capacity, the episomal nature of vector genomes, and the relatively low cost of vector production at high yields. However, while episomal HDAd vector genomes are lost after several rounds of cell division, they persist longer in slowly dividing and non-dividing cells (Figure 3F).^29^ Because HSCs are low proliferative, episomal HDAd5/35 genomes could express sgRNA and Cas9 for longer time periods. We show here that this contributes to toxicity in HSCs observed in progenitor colony-forming assays and after transplantation into NSG mice. However, CRISPR/Cas9 toxicity to HSCs is not only a problem associated with HDAd5/35^++^ delivery. Failure to successfully engraft in NSG mice was also observed with CRISPR/Cas9 that was co-delivered by electroporation of with plasmid-encoded CRISPR/Cas9.^30^ It is also noteworthy that in our studies we used CD34^+^ cells isolated by leukapheresis from granulocyte colony-stimulating factor (G-CSF) mobilized donors. While mobilized peripheral blood CD34^+^ cells is the main source for autologous HSC gene therapy, HSCs isolated from cord blood or fetal liver appeared to be more resistant to gene editing by endonucleases.^3^

Our data from HDAd-globin-CRISPR-1 transduction studies suggest that CRISPR/Cas9 toxicity is caused by nuclease-mediated DNA DSBs. It is well known that quiescent human HSCs are sensitized to apoptosis after DSB-inducing irradiation.^31^ In this context, we are currently testing whether HDAd5/35^++^ vectors expressing a hyper-accurate Cas9 variant (HypaCas9) that demonstrated fewer off-target DSBs than the Cas9 version used in our studies.^32^

**Figure 8:**
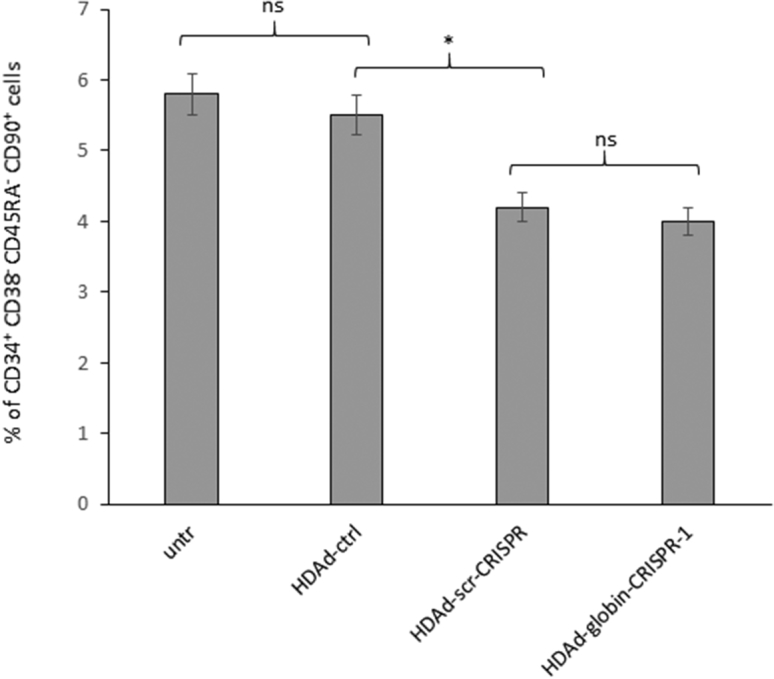
Effect of HDAd5/35^++^ Transduction on Phenotypically Primitive HSCs CD34^+^ cells were transduced with the indicated vectors at an MOI of 2,000 vp/cell, and the percentages of CD34^+^ CD38^−^ CD45RA^−^ CD90^+^ cells were measured 3 days later by flow cytometry. *p < 0.05.

However, because the HDAd-scr-CRISPR vector that is not able to stably bind the genomic DNA and cleave it also caused cytotoxicity to functional HSCs, we speculate that Cas9 interferes with cellular processes, possibly by binding to mRNA. Further mechanistic studies on the toxicity of the Cas9 protein or a non-binding sgRNA/Cas9 complex are required to consolidate our speculation. Interestingly, when we measured the percentage of CD34^+^/CD38^−^/CD45RA^−^CD90^+^ cells (a fraction highly enriched for HSCs) at day three after transduction, similar cytotoxicity was found for HDAd-globin-CRISPR-1 and HDAd-scr-CRISPR, suggesting that DSB-mediated CD34^+^ cell death might occur at later time points (Figure 8).

The aim of this study was to control the duration of CRISPR/Cas9 activity. For ZFN-based approaches, several techniques have been employed to limit ZFN expression levels and/or duration from transfected plasmids. Pruett-Miller et al.^33^ describe two techniques for ZFN regulation that allowed for reduction of ZFN cytotoxicity while maintaining its DNA cleavage activity. The authors first showed in 293 cells that ZFNs need to be expressed for only 32 hr after transfection to attain maximal gene targeting efficacy. To regulate ZFN half-life and the duration of expression, they first destabilized ZFNs by linking a ubiquitin moiety to the N terminus and regulated ZFN levels using a proteasome inhibitor. In a second approach, they destabilized ZFNs by linking a modified destabilizing FKBP12 domain to the N terminus and regulated ZFN levels by using a small molecule that blocks the destabilization effect of the N-terminal domain.^33^ Zhang et al.^34^ used a ZFN suicide strategy in the context of plasmids and showed lower ZFN-related toxicity in 293 cells. Inducible expression systems could potentially be used to regulate endonuclease expression. However, we previously found that, in the context of HDAd5/35 vectors, doxycycline (DOX) inducible Tet-on or autoregulated reverse tetracycline-controlled transactivator (rtTA) systems either displayed high levels of background expression without DOX (most likely because of activity of viral promoters present in the vector genome) or that the induction of the transgene was restricted to only a small subset of CD34^+^ cells.^11^

Here we employed the Acr peptides AcrIIA4 and AcrIIA2 to control CRISPR/Cas9 activity. In previous studies by Doudna’s group^19^ with anti-AcrII4-endcoding plasmids transfected prior to CRISPR/Cas9 delivery, reduction of off-target editing for CRISPRs designed for hemoglobin beta (HBB) and vascular endothelial growth factor (VEGF)-A genes was observed. We incorporated the Acr expression cassettes into HDAd5/35^++^ for efficient delivery in CRISPR/Cas9-expressing cells. We demonstrated in HUDEP-2 cells that co-infection with the CRISPR/Cas9 and Acr vectors decreased target site cleavage by 87%. The residual activity could be because of the possibility that not all HDAd-CRISPR-transduced cells were also transduced by the HDAd-Acr vector, or the possibility that Acr expression levels were not high enough to completely block Cas9 activity. In transduction and transplantation studies with CD34^+^ cells, we found that cells that were sequentially infected with HDAd-CRISPR and HDAd-Acr engrafted at a significantly higher rate. In contrast to transduction without HDAd-Acr, the frequency of CD34^+^ cells in engrafted human cells was not decreased. Target site disruption frequencies in engrafted human cells, measured 10 weeks after transplantation, were similar to those in pre-transplantation CD34^+^ cells, indicating that gene-edited primitive cells survived. Gene-edited human HSCs and progenitors were functional and after erythroid differentiation demonstrated increased γ-globin expression as a result of gene editing. Notably, in our studies, we did not pre-select gene-edited CD34^+^ cells before transplantation, as done in another study.^1^

Solving the problem of CRISPR/Cas9 toxicity to HSCs is not only relevant for γ-globin reactivation to treat hemoglobinopathies, but, in a larger context, for targeted integration, which is a major focus in clinical translation of HSC gene therapy approaches. Most of the approaches for targeted integration involve target site DSBs mediated by CRISPR/Cas9 to stimulate homology-directed repair pathways and gene addition using homologous donor templates.^35^, ^36^, ^37^, ^38^, ^39^

Cas9 and Acr peptides are bacterial proteins and therefore immunogenic. However, in the context of HSC gene therapy, this does not represent a problem because episomal HDAd vector genomes expressing these proteins are lost during HSC/progenitor cell division upon transplantation into lethally irradiated recipients. Furthermore, expression of immunogenic protein in lymphoid HSC progeny establishes tolerance, most likely through generation of tolerogenic T cells in the thymus.^40^, ^41^ Notably, direct *in vivo* gene transfer of CRISPR/Cas9 and Acr peptides most likely requires transient immunosuppression.^42^, ^43^

The HDAd5/35^++^ Acr vector tested in this study could also be a tool to reduce CRISPR/Cas9 toxicity to CD34^+^ cells after plasmid nucelofection. Considering a recent report on CRISPR/Cas9-mediated toxicity to human pluripotent stem cells, HDAd5/35^++^ Acr might also be relevant for genome editing in other human stem cells.^44^

## Materials and Methods

### Construction of HDAd Vectors

sgRNAs targeting the erythroid bcl11a enhancer (5′-CACAGGCTCCAGGAAGGGTT-3′), HBG1 promoter (5′-CAAGGCTATTGGTCAAGGCA-3′), as well as a non-targeting scrambled control sgRNA (5′-ATCGTTTCCGCTTAACGGCG-3′) were synthesized, annealed, and inserted into the BbsI site of pSPgRNA (Addgene, Cambridge, MA, USA), generating pSP-sgBCL11AE, pSP-sgHBG1, and pSP-sgScr, respectively. A Cas9 coding sequence amplified from pLentiCRISPRv2 (Addgene), U6sgRNA fragments released by BamHI digestion from pSPsgRNA constructs, and a previously described microRNA targeting region (miR-183/218)^14^ were sequentially cloned into the EcoRV-NotI, BamHI, and NotI sites of pBS-T-EF1α,^14^ forming pBST-sgBCL11AE-miR, pBST-sgHBG1-miR, and pBST-sgScr-miR. To obtain the recombinant adenoviral plasmids, we amplified 8-kb cassettes starting from the U6 promoter to the SV40 polyA signal sequence from the above pBST constructs and ligated them with NheI-XmaI digested pHCA by Gibson assembly (New England Biolabs), generating the corresponding pHCA-sgBCL11AE-miR, pHCA-sgHBG1-miR, and pHCA-sgScr-miR plasmids. For cloning of pHCA-Acr, a human codon-optimized AcrIIA4-T2A-AcrIIA2 sequence that was recently shown to inhibit SpCas9 activity^17^ was synthesized and cloned into the EcoRI site of pBS-Z-EF1α. Subsequently, the 3.5-kb EF1α-Acr-SV40 polyA cassette was amplified from pBS-Z-Acr and ligated with NheI-treated pHCA by Gibson Assembly. The correctness of all constructs was confirmed by HindIII and BamHI digestion and sequencing.

The construction of HDAd-ctrl, a HDAd5/35^++^ vector containing an EF1α-promoter GFP cassette, was described previously.^7^ First-generation Ad5/35^++^ vectors expressing GFP or mCherry were described previously.^14^

For the production of HDAd5/35^++^ vectors, corresponding plasmids were linearized with *PmeI* and rescued in 116 cells^45^ with AdNG163-5/35^++^, an Ad5/35^++^ helper vector containing chimeric fibers composed of the Ad5 fiber tail, the Ad35 fiber shaft, and the affinity-enhanced Ad35^++^ fiber knob.^7^ HD-Ad5/35^++^ vectors were amplified in 116 cells as described in detail elsewhere.^45^ Helper virus contamination levels were found to be <0.05%. Titers were 6–12 × 10^12^ vp/mL.

### CD34^+^ Cell Culture

CD34^+^ cells from G-CSF-mobilized adult donors were recovered from frozen stocks and incubated overnight in StemSpan H3000 (STEMCELL Technologies, Vancouver, BC, Canada) with penicillin and streptomycin (Pen/Strep), Flt3 ligand (Flt3L; 25 ng/mL), IL-3 (10 ng/mL), thrombopoietin (TPO) (2 ng/mL), and SCF (25 ng/mL). Cytokines and growth factors were from PeproTech (Rocky Hill, NJ, USA). (These cytokine and growth factor concentrations are 50% of those used for pre-stimulation of cells in the context of lentivirus transduction.) CD34^+^ cells were transduced with HDAd5/35^++^ vectors in low-attachment 12-well plates at MOIs indicated in the figure legends.

Differentiation of human HSCs into erythroid cells was done based on the protocol developed by Douay and Giarratana.^26^ In brief, in step 1, cells at a density of 10^4^ cells/mL were incubated for 7 days in Iscove’s modified Dulbecco’s medium (IMDM) supplemented with 5% human plasma, 2 IU/mL heparin, 10 μg/mL insulin, 330 μg/mL transferrin, 1 μM hydrocortisone, 100 ng/mL SCF, 5 ng/mL IL-3, 3 U/mL Epo, glutamine, and Pen-Strep. In step 2, cells at a density of 1 × 10^5^ cells/mL were incubated for 3 days in IMDM supplemented with 5% human plasma, 2 IU/mL heparin, 10 μg/mL insulin, 330 μg/mL transferrin, 100 ng/mL SCF, 3 U/mL Epo, glutamine, and Pen/Strep. In step 3, cells at a density of 1 × 10^6^ cells/mL were incubated for 8 days in IMDM supplemented with 5% human plasma, 2 IU/mL heparin, 10 μg/mL insulin, 330 μg/mL transferrin, 3 U/mL Epo, glutamine, and Pen/Strep. The total period of differentiation is 18 days (7 + 3 + 8 days).

### sgRNA/Cas9 Protein (RNP) or Plasmid Electroporation

Electroporation of CD34^+^ cells was carried out on an ECM 830 Square Wave Electroporation System (BTX, Holliston, MA, USA) according to manufacturer’s instructions. In brief, after recovery in culture medium described above for 48 hr, 2 × 10^5^ CD34^+^ cells were washed once with Ca^2+^/Mg^2+^-free PBS and resuspended in 100 μL of BTXpress electroporation solution. Then, cells were mixed with 16 μg of Cas9 protein (PNA Bio) pre-coupled with sgRNA (globin-CRISPR-1) at a molar ratio of 1:5 or 1 μg of pmaxGFP plasmid (Lonza, Basel, Switzerland), transferred into a 0.2-cm cuvette and electroporated at 250 V for 5 μs. After electroporation, the cells were incubated in culture medium for 18 hr for apoptosis analyses. The sgRNA was *in vitro* transcribed with the mMESSAGE Machine T7 Ultra kits (Thermo Fisher Scientific), according to the manufacturer’s instructions.

### HUDEP-2 Cells/Erythroid Differentiation

HUDEP-2 cells^28^ were cultured in StemSpan SFEM medium (STEMCELL Technologies) supplemented with 100 ng/mL SCF, 3 IU/mL EPO, 10^−6^ M dexamethasone, and 1 μg/mL DOX. Erythroid differentiation was induced in IMDM containing 5% human AB serum, 100 ng/mL SCF, 3 IU/mL EPO, 10 μg/mL insulin, 330 μg/mL transferrin, 2 U/mL heparin, and 1 μg/mL DOX for 6 days.

### CFU Assay

CFU assays with transduced CD34^+^ cells were performed using ColonyGEL (Reachbio, Seattle, WA, USA) with human complete medium according to the manufacturer’s protocol. Colonies were scored 14 days after plating.

### Magnetic-Activated Cell Sorting

Anti-human CD45-conjugated microbeads were from Miltenyi Biotech (Bergisch Gladbach, Germany). Cell purification was performed according to the manufacturer’s protocol.

### T7E1 Mismatch Nuclease Assay

Genomic DNA was isolated using PureLink Genomic DNA Mini Kit per provided protocol (Life Technologies, Carlsbad, CA, USA).^46^ Genomic segments encompassing the targeted site of erythroid bcl11a enhancer or HBG1/2 promoter were amplified by PCR primers: BCL11A forward, 5′-AGAGAGCCTTCCGAAAGAGG-3′, reverse, 5′-GGCAGCTAGACAGGACTTGG-3′; HBG1/2 forward, 5′-CAGGGTTTCTCCTCCAGCATCTTCCACAT-3′, reverse, 5′-AGCAGCAGTATCCTCTTGGGG-3′. PCR products were hybridized and treated with 2.5 U of T7EI (NEB) for 20 min at 37°C. Digested PCR products were resolved by 10% Tris/borate/EDTA buffer (TBE) PAGE (Bio-Rad) and stained with ethidium bromide. 100 bp DNA Ladder (New England Biolabs) was used. Band intensity was analyzed using ImageJ software. Percentage cleavage = (1 − sqrt(parental band/(parental band + cleaved bands)) × 100%.

### Flow Cytometry

Cells were resuspended at 1 × 10^6^ cells/100 μL in PBS supplemented with 1% fetal calf serum (FCS) and incubated with FcR blocking reagent (Miltenyi Biotech, Auburn, CA, USA) for 10 min on ice. Next the staining antibody solution was added in 100 μL per 10^6^ cells and incubated on ice for 30 min in the dark. After incubation, cells were washed once in fluorescence-activated cell sorting (FACS) buffer (PBS, 1% fetal bovine serum [FBS]). For secondary staining the staining step was repeated with a secondary staining solution. After the wash, cells were resuspended in FACS buffer and analyzed using an LSRII flow cytometer (BD Biosciences, San Jose, CA, USA). Debris was excluded using a forward scatter-area and sideward scatter-area gate. Single cells were then gated using a forward scatter-height and forward scatter-width gate. Flow cytometry data were then analyzed using FlowJo (version 10.0.8; FlowJo). Antibodies from Miltenyi Biotec included anti-human CD45-allophycocyanin (APC) (clone: 5B1), anti-human CD3-PE/Cy7 (clone: REA613), anti-human CD34-PE (clone: AC136), and anti-human Glycophorin A-VioBlue (clone: REA175). Anti-human CD19-PE (clone: HIB19), anti-human CD33-BV421 (WM53), anti-human CD38-PercP/Cy5.5 (clone: HIT2), and anti-human CD90-BV605 (clone: 5E10) were from BD Biosciences. Anti-human CD45RA-APC/Cy7 (clone: HI100) and Annexin V-PE apoptosis kit with 7-AAD were from BioLegend (San Diego, CA, USA).

### Intracellular Flow Cytometry Detecting Human γ-Globin Expression

The FIX & PERM cell permeabilization kit (Thermo Fisher Scientific) was used and the manufacturer’s protocol was followed. In brief, ∼1 × 10^6^ cells were resuspended in 100 μL of FACS buffer (PBS supplemented with 1% FCS); 100 μL of reagent A (fixation medium) was added and incubated for 2–3 min at room temperature; and 1 mL of pre-cooled absolute methanol was then added, mixed, and incubated on ice in the dark for 10 min. The samples were then washed with FACS buffer and resuspended in 100 μL of reagent B (permeabilization medium) and 1 μg of hemoglobin γ antibody (catalog [cat] #sc-21756 PE; Santa Cruz Biotechnology), and incubated for 30 min at room temperature. After the wash, cells were resuspended in FACS buffer and analyzed.

### Globin HPLC

Individual globin chain levels were quantified on a Shimadzu Prominence instrument with an SPD-10AV diode array detector and an LC-10AT binary pump (Shimadzu, Kyoto, Japan). Vydac 214TP C4 Reversed-Phase columns for polypeptides (214TP54 Column, C4, 300 Å, 5 μm, 4.6 mm intradermal [i.d.] × 250 mm) (Hichrom, UK) were used. A 40%–60% gradient mixture of 0.1% trifluoroacetic acid in water and acetonitrile was applied at a rate of 1 mL/min.

### qPCR for Ad Genome and Cas9 mRNA

For absolute quantification of adenoviral genome copies per cell, genomic DNA was isolated from cells using PureLink Genomic DNA Mini Kit per provided protocol (Life Technologies) and used as template for qPCR performed using the power SYBR green PCR master mix (Thermo Fisher Scientific). Cas9 forward, 5′-ATCCTGACCTTCCGCATCCCCT-3′, and reverse, 5′-CGCCCTTGTCCACCACTTCCTC-3′, were used as primers. Each condition was run in triplicates. Serial dilutions of purified HDAd-globin-CRISPR-1 viral DNA were used as a standard curve. Data were presented as viral copy number per cell.

For comparative quantification of Cas9 and globin gene transcription, total RNA was extracted from cells by using TRIzol reagent (Thermo Fisher Scientific) following the manufacturer’s phenol-chloroform extraction method. cDNA was generated by using QuantiTect Reverse transcription kit (QIAGEN, Germantown, MD, USA), and later used as template for real-time PCR using the same kit described as above. In addition to the Cas9 primers shown above, the following primers were used: human γ-globin forward, 5′-GTGGAAGATGCTGGAGGAGAAA-3′, and reverse, 5′-TGCCATGTGCCTTGACTTTG-3′; human β-globin forward, 5′-CTCATGGCAAGAAAGTGCTCG-3′, and reverse, 5′-AATTCTTTGCCAAAGTGATGGG-3′; GAPDH (internal control) forward, 5′-GGCCTCCAAGGAGTAAGACC-3′, and reverse, 5′-AGGGGTCTACATGGCAACTG-3′. PCR was performed on a StepOnePlus real-time PCR system (Applied Biosystems, Foster City, CA, USA). Results were calculated by the comparative cycle threshold (Ct) method using the formula: 2^ΔΔCt^.

### Animals

All experiments involving animals were conducted in accordance with the institutional guidelines set forth by the University of Washington. The University of Washington is an Association for the Assessment and Accreditation of Laboratory Animal Care International (AALAC)-accredited research institution, and all live animal work conducted at this university is in accordance with the Office of Laboratory Animal Welfare (OLAW) Public Health Assurance (PHS) policy, US Department of Agriculture (USDA) Animal Welfare Act and Regulations, the Guide for the Care and Use of Laboratory Animals, and the University of Washington’s Institutional Animal Care and Use Committee (IACUC) policies. The studies were approved by the University of Washington IACUC (Protocol No. 3108-01). The immunodeficient NSG mice were obtained from the Jackson Laboratory (Bar Harbor, ME, USA). For HSC transplantation, NSG recipient mice received 300 rad whole-body irradiation. 2.5 × 10^5^ whole bone marrow cells of non-irradiated NOG mice were mixed with 6 × 10^5^ transduced human CD34^+^ cells and injected intravenously into recipient mice at 4 hr post-irradiation.

### Statistical Analyses

For comparisons of multiple groups, one-way and two-way ANOVA with Bonferroni post-testing for multiple comparisons was employed. Statistical analysis was performed using GraphPad Prism version 6.01 (GraphPad Software, La Jolla, CA, USA).

## Author Contributions

A.L. provided the conceptual framework for the study. C.L., N.P., and A.L. designed the experiments. C.L., N.P., S.G., and H.W. performed the experiments. T.P. provided critical comments on the manuscript. A.L. wrote the manuscript.
